# Development of an interactive web-based tool to conduct and interrogate meta-analysis of diagnostic test accuracy studies: MetaDTA

**DOI:** 10.1186/s12874-019-0724-x

**Published:** 2019-04-18

**Authors:** Suzanne C. Freeman, Clareece R. Kerby, Amit Patel, Nicola J. Cooper, Terry Quinn, Alex J. Sutton

**Affiliations:** 1 NIHR Complex Reviews Support Unit, University of Leicester & University of Glasgow, Glasgow, UK; 2 Biostatistics Research Group, Department of Health Sciences, University of Leicester, Leicester, LE1 7RH UK; 3 Institute of Cardiovascular and Medical Sciences, University of Glasgow, Glasgow, G12 8QQ UK; 4 Cochrane Dementia and Cognitive Improvement Group, Oxford, UK

**Keywords:** Diagnostic test accuracy, Meta-analysis, Application

## Abstract

**Background:**

Recommended statistical methods for meta-analysis of diagnostic test accuracy studies require relatively complex bivariate statistical models which can be a barrier for non-statisticians. A further barrier exists in the software options available for fitting such models. Software accessible to non-statisticians, such as RevMan, does not support the fitting of bivariate models thus users must seek statistical support to use R, Stata or SAS. Recent advances in web technologies make analysis tool creation much simpler than previously. As well as accessibility, online tools can allow tailored interactivity not found in other packages allowing multiple perspectives of data to be displayed and information to be tailored to the user’s preference from a simple interface. We set out to: (i) Develop a freely available web-based “point and click” interactive tool which allows users to input their DTA study data and conduct meta-analyses for DTA reviews, including sensitivity analyses. (ii) Illustrate the features and benefits of the interactive application using an existing DTA meta-analysis for detecting dementia.

**Methods:**

To create our online freely available interactive application we used the existing R packages lme4 and Shiny to analyse the data and create an interactive user interface respectively.

**Results:**

MetaDTA, an interactive online application was created for conducting meta-analysis of DTA studies. The user interface was designed to be easy to navigate having different tabs for different functions. Features include the ability for users to enter their own data, customise plots, incorporate quality assessment results and quickly conduct sensitivity analyses. All plots produced can be exported as either .png or .pdf files to be included in report documents. All tables can be exported as .csv files.

**Conclusions:**

MetaDTA, is a freely available interactive online application which meta-analyses DTA studies, plots the summary ROC curve, incorporates quality assessment results and allows for sensitivity analyses to be conducted in a timely manner. Due to the rich feature-set and user-friendliness of the software it should appeal to a wide audience including those without specialist statistical knowledge. We encourage others to create similar applications for specialist analysis methods to encourage broader uptake which in-turn could improve research quality.

## Background

### Background to meta-analysis of diagnostic test accuracy studies

Diagnostic tests are routinely used in healthcare settings for confirming or excluding diagnoses. They generally comprise of a measure which splits individuals into healthy or diseased. Diagnostic tests in primary care can generally be done on any patient and are normally quick and painless. In hospital settings, diagnostic tests can be more invasive and expensive, and are often only carried out on specific populations of individuals where the tests may be necessary to determine treatment pathways [1]. Diagnostic tests have been around for a long time. However, as our understanding of biology and disease has increased, along with advances in technology, many new diagnostic tests have emerged and there is now a plethora of diagnostic tests available [1]. For example, with some conditions, such as cancer, studies aim to identify a new diagnostic test that is still as accurate as the standard test, yet less costly or invasive. Diagnostic tests are rarely 100% accurate so rigorous testing is required [1]. There can be many aspects to evaluating a diagnostic test including ability to measure the desired parameter, cost-effectiveness and accuracy. In this paper we focus on assessing the accuracy of diagnostic tests.

To assess accuracy, a diagnostic test is compared to the “gold standard” test which is assumed to provide the true diagnosis of individuals. The value used to split the population into healthy or diseased is known as the threshold or cut-off value. The results of a diagnostic test are often reported in a 2 × 2 table, as in Table 1. The true positive (TP) rate is the number of patients correctly identified as having the disease by the diagnostic test. The true negative (TN) rate is the number of patients correctly identified as not having the disease by the diagnostic test. The false positive (FP) rate is the number of patients who do not have the disease but have a positive test result. The false negative (FN) rate is the number of patients who have the disease but have a negative test result. There are two parameters which are often used to assess the accuracy of diagnostic tests; sensitivity and specificity. Sensitivity is the ability of the diagnostic test to correctly identify patients with the disease amongst patients who have the disease (i.e. TP/TP + FN). Specificity is the ability of the diagnostic test to correctly identify the healthy individuals amongst patients who do not have the disease (i.e. TN/FP + TN).

**Table 1:** Illustration of a 2 × 2 table of diagnostic test results

		Gold standard	
		Positive	Negative
Index test	Positive	True Positives (TP)	False Positives (FP)
	Negative	False Negatives (FN)	True Negatives (TN)

A meta-analysis (MA) of diagnostic test accuracy (DTA) studies synthesises both sensitivity and specificity from multiple studies to evaluate the performance of a diagnostic test. DTA MA should take into account the correlation between sensitivity and specificity and is often performed using either the bivariate or hierarchical summary receiver operating characteristic (HSROC) models, and the results presented either around a mean accuracy point or as a summary receiver operating characteristic (SROC) curve [2–4]. The SROC curve plots sensitivity on the y-axis against 1-specificity on the x-axis illustrating how sensitivity and specificity vary for different thresholds of a test.

### Static versus interactive graphs

Conventionally, SROC curves are published as static graphs. Static graphs can be limiting as they must display all of their elements on the same surface at the same time. Otherwise, to represent all the dimensions of the data and illustrate the necessary perspectives, multiple static graphs must be produced [5]. An alternative to static graphs is to consider interactive graphs. Interactivity allows multiple perspectives of the data to be seen and can be made up of layers, allowing one space to be used for describing multiple types of analyses where each layer is only visible when the user selects it [6]. Importantly, interactivity allows users to explore the data themselves and can provide a useful tool to aid sensitivity analyses. A trade-off exists between static and interactive graphs between the time it takes to generate the new image and the space needed to present the many static graphs necessary to illustrate the same point [6].

### Why is an interactive online application needed?

Cochrane publish a number of diagnostic test accuracy reviews each year. A search of the Cochrane Database of Systematic Reviews on the 7th September 2018 identified 99 reviews of type ‘diagnostic’ published between October 2009 and August 2018. However, the Cochrane software RevMan [7] uses the Moses-Littenburg method [8, 9] for DTA MA which does not properly take into account random effects and the correlation between sensitivity and specificity [10]. Therefore, using bivariate or HSROC models is more appropriate for MA of DTA studies [2]. To properly conduct DTA MA in RevMan other programs such as Stata, SAS, R or WinBUGS are needed to conduct the statistical analysis and then the HSROC parameters are ‘fed back’ into RevMan to produce SROC plots. Furthermore, feedback from Cochrane review teams highlighted frustrations with the complexity of existing DTA MA software approaches. We set out to develop an interactive application which could be used by both researchers familiar with the DTA MA process, but who don’t necessarily have the statistical expertise to use specialist software such as Stata or R, and statisticians, to allow a comprehensive analysis of their data and publication quality figures to be conducted in a single package.

Sensitivity analysis is an essential part of any statistical analysis. It allows examination of both data and assumptions. In MA sensitivity analysis is often conducted excluding any particularly large or extreme studies, or studies deemed to be of low quality to assess the robustness of the parameter estimates from the primary analysis. In software such as RevMan excluding one trial involves running a new analysis. We set out to encourage sensitivity analyses within the application by allowing trials to be excluded in an easy interactive manner.

A 2008 review investigating how diagnostic information was graphically presented concluded that often multiple graphs are needed to in order to provide both a detailed overview of the results and to communicate the information needed to inform clinical practice [11]. Furthermore, effective interactive tools need to have appropriate statistical functioning, alongside high quality graphics to provide a pleasant experience for the user [12]. We considered graphical design alongside statistical analysis to develop a user-friendly intuitive application allowing users to explore their data, conduct sensitivity analyses and assess the impact of assumptions on the parameter estimates.

### Objectives

We set out to develop a freely available web-based “point and click” interactive online application which allows users to input their own data and conduct meta-analyses of diagnostic test accuracy study data including sensitivity analyses. We illustrate the benefits of the interactive application using an existing DTA MA on assessing protein in cerebrospinal fluid to identify patients with mild cognitive impairment who would develop dementia.

#### Implementation

We built MetaDTA, an interactive application to facilitate analysis and aid understanding for researchers, clinicians and students, focusing on the SROC plot.

### Software

To create our online freely available interactive application we used the statistical software R [13] and the existing packages Shiny [14] and lme4 [15]*.* Shiny is a package that allows R users to create web applications with interactive user interfaces without prior knowledge of web development languages such as HTML, JavaScript and CSS [14]. lme4 is a package that fits generalised linear mixed effect models [15]. MetaDTA is hosted on the shinyapps server which makes it available to any user with a web browser, without requiring any specialist statistical software. The application is available at https://crsu.shinyapps.io/dta_ma/.

### Statistical analysis

The random effects bivariate binomial model of Chu & Cole [4] is fitted as a generalised linear mixed effect model using the glmer function from the package lme4 [15, 16]. Sensitivity and specificity are jointly modelled and the estimates from each study are assumed to vary but come from a common underlying distribution with an unstructured between-study covariance matrix [16].

The bivariate model has been shown to be mathematically identical to the HSROC model [17]. Therefore the HSROC parameters are estimated using the bivariate model parameters and the equivalence equations of Harbord et al. [17]. The SROC plot is drawn using the resulting HSROC parameters. Positive and negative likelihood ratios and the diagnostic odds ratio are calculated directly from the estimates of logit sensitivity and logit specificity. The R package msm is used to implement the delta method to calculate the standard errors of the likelihood ratios and diagnostic odds ratio to allow calculation of the confidence intervals [18].

Data on the quality of the primary diagnostic accuracy studies, evaluated using the QUADAS-2 tool [19], can be incorporated within SROC plots in MetaDTA. The QUADAS-2 tool consists of four domains i) patient selection, ii) index test, iii) reference standard, and iv) flow of patients through the study and timing of the index test(s) and reference standard. All domains are assessed in terms of risk of bias, and domains i) to iii) in terms of concerns regarding applicability to the review question [19].

### User Interface

The user interface was designed to be user-friendly and intuitive and follows the process of conducting an analysis: Load Data, Meta-Analysis and Sensitivity Analysis. All pages have been constructed with a sidebar on the left displaying the options available for the user to select. A fourth page, References, includes some of the key references for the statistical methods and default datasets used within MetaDTA.

**Fig. 1:**
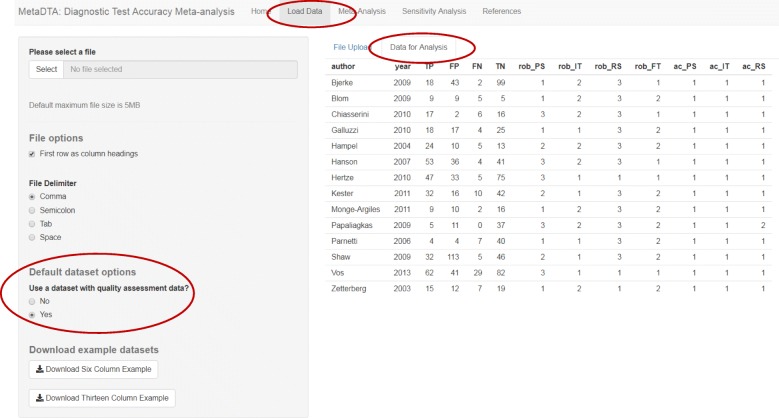
Data for Analysis tab on the Load Data page. Data displayed is from the inbuilt dataset on assessing dementia

The Load Data page offers users the option to upload their own dataset in either a six column format or in a thirteen column format in which the additional seven columns contain quality assessment results from QUADAS-2. The Load Data page is pre-loaded with two inbuilt datasets: unhealthy alcohol use [20] and dementia [21]. The unhealthy alcohol use dataset is the default dataset and is in the six column format. The dementia dataset contains quality assessment results and can be accessed by selecting ‘Yes’ to the question ‘Use a dataset with quality assessment data?’ under the ‘Default dataset options’ on the sidebar. Default datasets are overwritten when users choose to upload their own dataset. The tab ‘Data for Analysis’ will always display the dataset being analysed (Fig. 1).

**Fig. 2:**
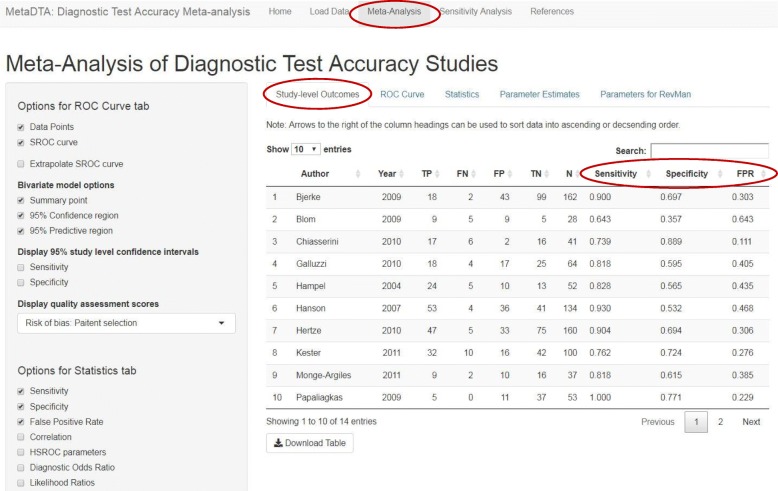
Study-level Outcomes tab on the Meta-Analysis page. Estimates of sensitivity, specificity and false positive rate for each trial

The Meta-Analysis page consists of five tabs: Study-level Outcomes, ROC curve, Statistics, Parameter Estimates and Parameters for RevMan. The ‘Study-level Outcomes’ tab presents estimates of the sensitivity, specificity and false-positive rate for each trial (Fig. 2). Throughout MetaDTA all tables can be downloaded as .csv files and all figures can be downloaded as either .png or .pdf files.

**Fig. 3:**
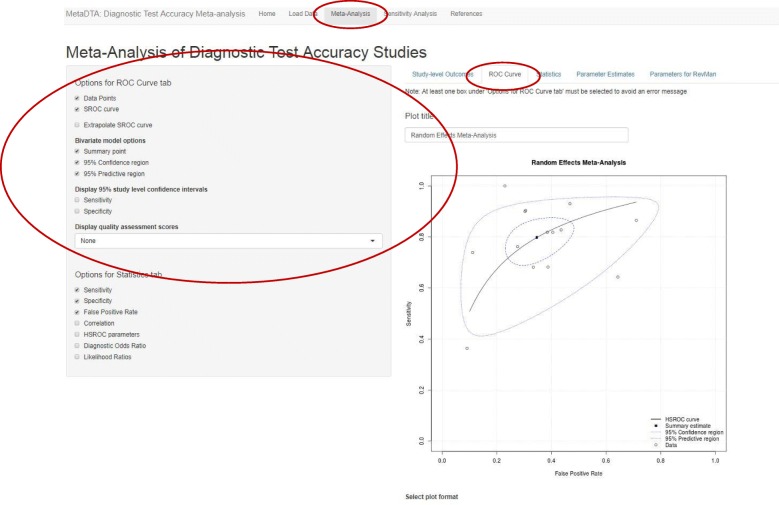
ROC curve tab on Meta-Analysis page. ROC curve showing data points, summary estimate, 95% confidence region, 95% predictive region and HSROC curve for all trials. Data points have been coloured according to the patient selection domain for risk of bias from the QUADAS-2 tool. The sidebar on the left shows the options available to customise the plot

On the ‘ROC curve’ tab users are able to choose whether to display the data point from each trial, the SROC curve, summary point, 95% confidence region, 95% predictive region and 95% confidence intervals for sensitivity and specificity of each data point. When SROC curve is selected users may also choose to extrapolate the SROC curve. Users are able to specify their own title for the plot. Additional functionality includes displaying the sensitivity and false-positive rate below the graph for the appropriate study when a data point is clicked on. For datasets in the thirteen column format an additional drop down menu contains the individual domains from the QUADAS-2 tool. When a domain is selected the data points on the ROC plot are coloured dependent on their quality assessment score: green for low risk of bias/applicability, red for high risk of bias/applicability and grey for unclear (Fig. 3). Choosing one of two further options in the drop down menu, ‘Risk of bias (all)’ or ‘Applicability concerns (all)’, will display pie charts on the ROC plot summarising all domains of QUADAS-2 concerning either risk of bias or applicability respectively. In addition, when either of these options is chosen and the user clicks on the middle of the pie chart for a particular study a larger version of the pie chart is displayed below the ROC plot.

**Fig. 4:**
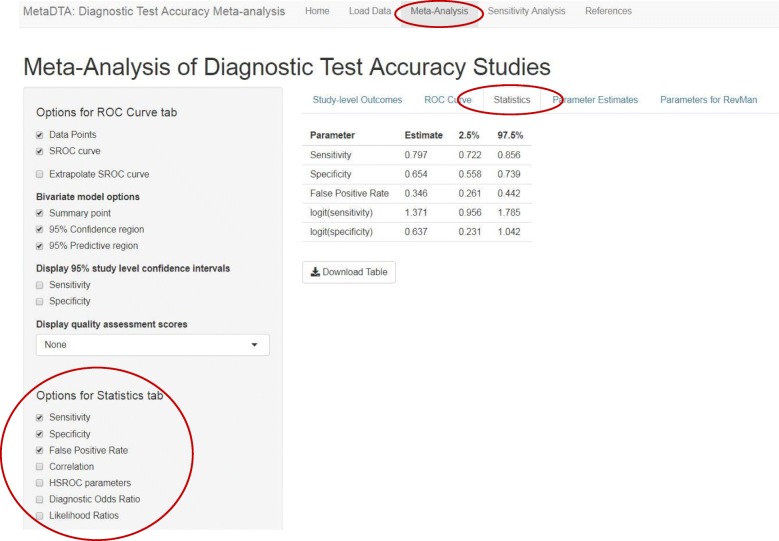
Statistics tab on the Meta-Analysis page. Table shows estimates of sensitivity, specificity and false positive rate across all trials. The sidebar on the left shows the options available to customise the table

**Fig. 5:**
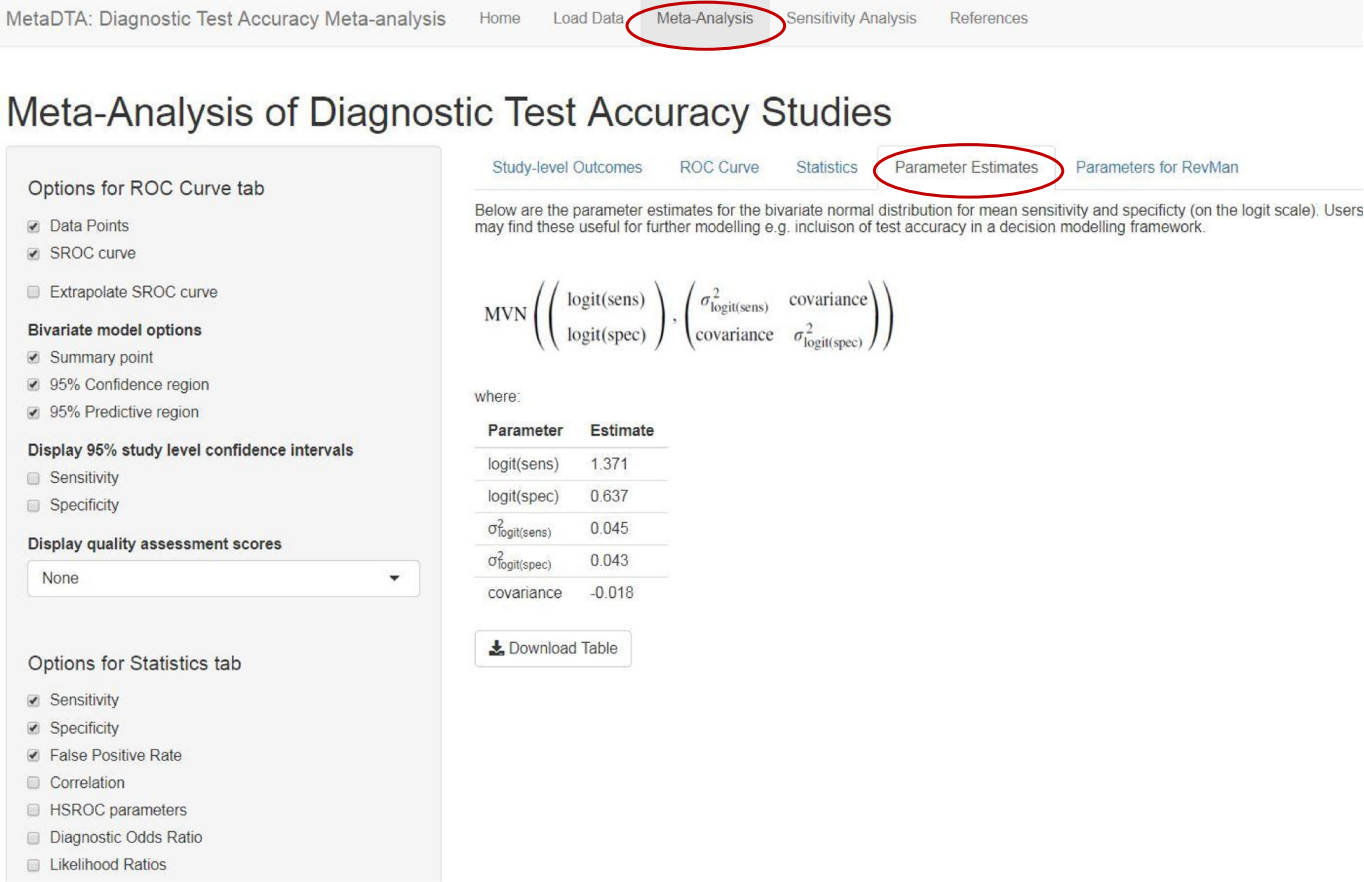
Parameter Estimates tab on the Meta-Analysis page. Table shows parameter estimates from the bivariate normal distribution for mean sensitivity and specificity (on the logit scale) which may be useful for further modelling

**Fig. 6:**
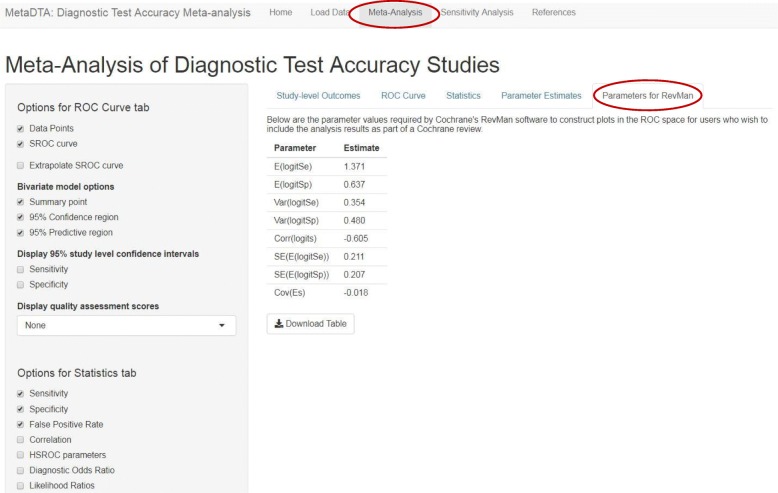
Parameters for RevMan tab on the Meta-Analysis page. Table shows parameter values require by RevMan to construct plots in the ROC space

The ‘Statistics’ tab tabulates point estimates and 95% confidence intervals for the statistics selected by the user from the list in the sidebar on the left (Fig. 4). The ‘Parameter Estimates’ tab provides the bivariate normal distribution for mean sensitivity and specificity on the logit scale which may be useful for further modelling such as the inclusion of test accuracy in a decision modelling framework (Fig. 5). The ‘Parameters for RevMan’ tab provides the parameter values required by RevMan to allow construction of plots in the ROC space for users who wish to include the analysis results as part of a Cochrane review (Fig. 6).

**Fig. 7:**
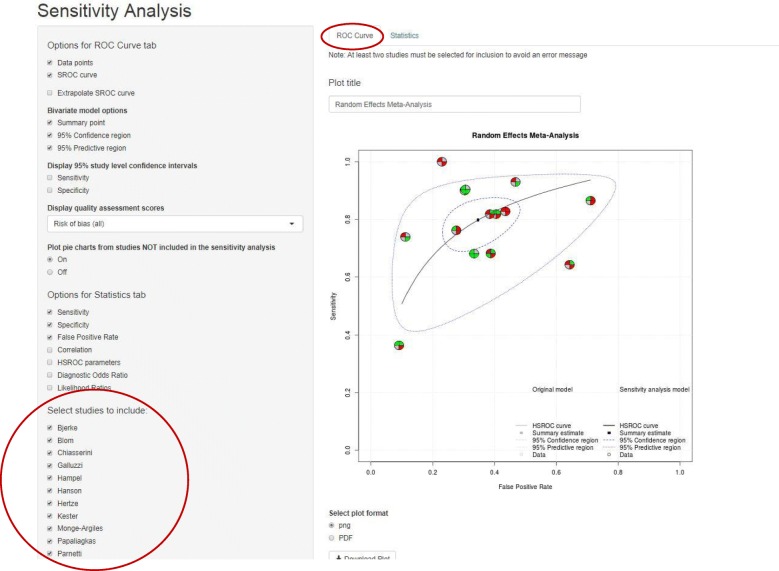
ROC curve tab on the Sensitivity-Analysis page. ROC curve showing data points, summary estimate, 95% confidence region, 95% predictive region and HSROC curve for all trials excluding Papaliagkas. Analysis of all trials is shown in grey. Data points are displayed as pie charts summarising the risk of bias domains from the QUADAS-2 tool

**Fig. 8:**
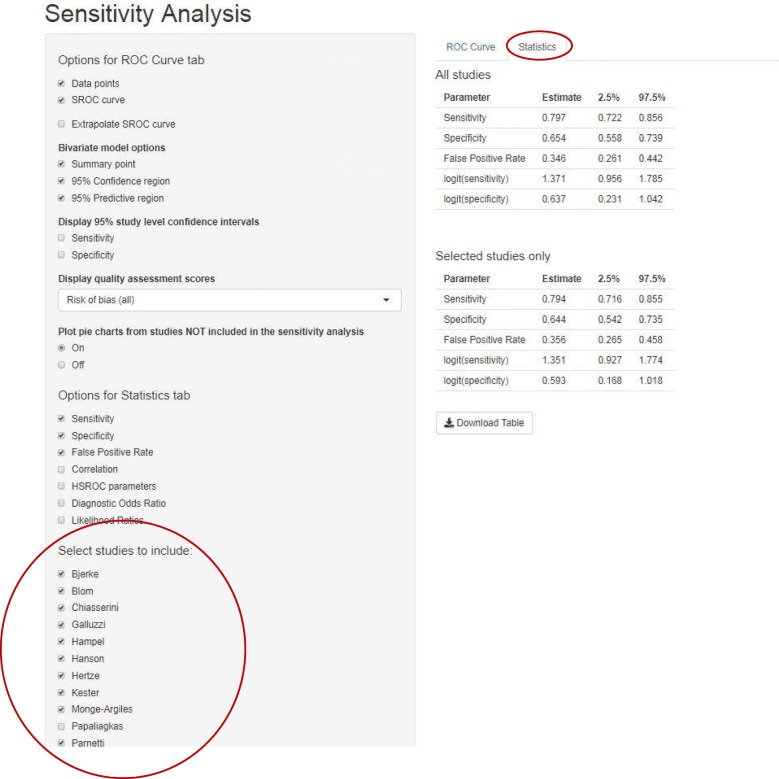
Statistics tab on the Sensitivity Analysis page. Top table shows estimates of sensitivity, specificity and false positive rate across all trials. Bottom table shows estimates of sensitivity, specificity and false positive rate across all trials excluding Gomez and Gordon

The Sensitivity Analysis page is similar in format to the Meta-Analysis page, with an additional option in the sidebar on the left which allows users to select which studies they would like to include in their sensitivity analysis. The ‘ROC curve’ tab displays in grey estimates from studies included in the main analysis but excluded from the sensitivity analysis. The overall pooled estimate from the main analysis is also displayed in grey. Estimates from the studies included in the sensitivity analysis are displayed in black with the overall pooled estimate from these studies in blue. For datasets in the thirteen column format, if selected as an option, pie charts summarising risk of bias or applicability concerns are displayed in grey for studies excluded from the sensitivity analysis (Fig. 7). The ‘Statistics’ tab displays two tables: estimates from the sensitivity analysis and estimates from the analysis of all trials (Fig. 8).

## Results

### Illustrative example using dementia data

The dataset used to illustrate MetaDTA is taken from a meta-analysis assessing protein in cerebrospinal fluid to identify patients with mild cognitive impairment who would develop Alzheimer’s disease dementia or other forms of dementia [21]. The dataset consists of fourteen studies. Figure 2 shows the estimates of sensitivity, specificity and false-positive rate from each trial.

Across the fourteen studies the pooled estimate of sensitivity was 79.7% (95% confidence interval (CI): 72.2, 85.6%) and the pooled estimate of specificity was 65.4% (95% CI: 55.8, 73.9%) (Figs. 3 & 4). From Fig. 2 it can be seen that the Papaliagkas study has zero false negative observations giving a sensitivity equal to 1. We chose to conduct a sensitivity analysis to determine how influential this perfect sensitivity is on the mean estimates. Removing the Papaliagkas study had little effect on the pooled estimates of sensitivity and specificity. The pooled estimate of sensitivity reduced slightly to 79.4% (95% CI: 71.6, 85.5%) whilst the false positive rate reduced slightly to 64.4% (95% CI, 54.2, 73.5%) (Figs. 7 & 8).

### Decision making context

In England and Wales, the National Institute for Health and Care Excellence (NICE) are responsible for determining which treatments are available on the NHS. Evidence synthesis feeds into economic evaluations which inform clinical decision-making by NICE. Bujkiewicz et al. demonstrated the feasibility and usefulness of interactive applications during a NICE Technology Appraisal meeting [22]. They developed an interactive Excel application, Transparent Interactive Decision Interrogator (TIDI), to allow for real-time sensitivity analyses which was used during a NICE Technology Appraisal meeting [22]. An Excel-based interface was constructed including graphical controls which allowed a range of assumptions to be explored. Statistical analyses were conducted ‘behind the scene’ using R and WinBUGS. Whilst the Excel-based interface provided a familiar user-interface, the application required the installation of both R and WinBUGS on the computer being used. Furthermore, the application required statistical expertise as re-programming was needed for each new dataset. However, a survey of committee members following the meeting found that the application was viewed in a positive manner providing support to decision makers by allowing a range of scenarios and assumptions to be explored in real time and speeding up the decision making process [22].

An advantage of MetaDTA is that, unlike TIDI, it does not require knowledge of any specialist statistical software packages such as Stata, R or WinBUGS to use. Furthermore, MetaDTA has the potential to be developed further to aid in the decision making process. MetaDTA already provides the estimated parameters of the bivariate normal distribution required for probabilistic sampling for stochastic decision modelling based on the correlated mean estimates of accuracy from the meta-analysis.

Further work could include the development of an interface between MetaDTA and associated economic decision models which could be used within meetings to conduct real-time sensitivity analyses on a range of scenarios and assumptions. Countries across the world are using and/or developing their own formal decision making processes (e.g. Canada, Australia, Brazil). Therefore, the need for an online application to conduct real-time analyses will continue to increase as the number of countries adopting formal decision making processes increases. Creating and sharing online resources such as this app, which can be used worldwide with just an internet browser, may minimise duplication and effort across countries.

## Discussion

We created MetaDTA, an online interactive ‘point-and-click’ application for meta-analysis of DTA studies. MetaDTA allows users to upload their own data, analyses the data and presents the results in downloadable formats. We developed a user-friendly intuitive user interface which allows users to explore their data and the analysis. The application uses the statistically rigorous bivariate model to analyse the data and much of the data and results can be presented graphically via an interactive SROC plot. There are many options available allowing users to customise the graphs to suit their needs including the option to incorporate quality assessment results from the QUADAS-2 tool. In addition, all statistical parameter estimates are presented in tables with uncertainty. All tables and figures can be downloaded from the application. Furthermore, we encourage the use of sensitivity analyses by allowing users the option to remove trials from the analysis.

We believe that sensitivity analysis is the key to ensuring that an analysis is robust. However, some published meta-analyses of DTA studies may hypothesise about how their results could be affected by certain studies but don’t always conduct sensitivity analyses. Sensitivity analyses can be time-consuming and may involve ‘going back to the start’ and creating a new dataset excluding influential studies and running the analysis again. MetaDTA avoids this allowing users to remove a study through one click of a button and presenting the results alongside the original analysis so that the impact of removing a study can be seen visually in both figures and Tables. A key element of sensitivity analyses is to assess the impact of study quality on the DTA MA results. MetaDTA allows the results of quality assessment from the QUADAS-2 tool to be included in the uploaded data file. An assessment of how the DTA MA results change when low quality studies are excluded can then be easily conducted by removing the low quality studies with results displayed visually in both figures and tables. We believe that MetaDTA which allows customisable and statistically informative graphics could improve the conduct of sensitivity analyses.

MetaDTA was developed by statisticians at the UK National Institute of Health Research Complex Reviews Support Unit (CRSU) with direct input from end-users. MetaDTA was first presented at a CRSU workshop in April 2018. Feedback from the meeting was positive and requests for additional functionality, such as estimates for the HSROC model, were incorporated into the application. We are aware of several other standalone R packages which can be used to conduct the statistical analyses required to meta-analyse DTA studies [16]. However, we are only aware of one R package which both fits a bivariate model to synthesise DTA studies and provides a user interface [23]. The package meta4diag fits a Bayesian bivariate normal model and provides an interactive graphical interface so that the full functionality of the package can be accessed without requiring any R programming. However, meta4diag requires users to have R installed on their device and the results are presented as R output requiring the user to have some R knowledge. We believe MetaDTA has advantages over meta4diag as the only software needed is an internet browser and that the direct input from end-users has resulted in an application that is truly user-friendly for both statisticians and non-statisticians. As with all forms of statistical analysis, we encourage users unfamiliar with the statistical methodology to seek statistical support to ensure correct interpretation of the results.

Building on the concept of explorable explanations first proposed by Bret Victor in 2011 [24] a further advantage of this application is the potential to encapsulate a specific dataset within the application. For example, as part of the online supplementary material for a journal paper a link could be placed which when followed takes the user to a version of this application which contains the data reported in the journal paper. Explorable explanations enable and encourage the reader to become an active participant in the learning process allowing text to be used as an environment to think in [25]. In this case, the user would be able to explore the dataset themselves, repeat the analyses reported in the paper, assess the impact of modelling assumptions and conduct their own sensitivity analyses scrutinising any concerns they may have.

## Conclusion

We built a freely available interactive online application which meta-analyses DTA studies, produces the SROC plot, incorporates quality assessment results and allows for sensitivity analyses to be conducted in a timely manner. MetaDTA will allow a wide range of users to carry out specialised analyses without needing software beyond an internet browser. We encourage others to create similar applications for specialist analysis methods to encourage broader uptake which in-turn could improve research quality.

## Availability and requirements

**Project name:** MetaDTA **(**Diagnostic Test Accuracy Meta-Analysis).

**Project home page:** https://crsu.shinyapps.io/dta_ma/

**Operating system(s):** Platform independent.

**Programming language:** R.

**Other requirements:** Internet browser.

**License:** Not applicable.

**Any restrictions to use by non-academics:** None.
